# Relation of Overweight/Obesity to Reward Region Response to Food Reward and the Moderating Effects of Parental History of Eating Pathology in Adolescent Females

**DOI:** 10.3390/nu15112558

**Published:** 2023-05-30

**Authors:** Sonja Yokum, Eric Stice

**Affiliations:** 1 Oregon Research Institute, Springfield, OR 97477, USA; 2 Department of Psychiatry and Behavioral Sciences, Stanford University School of Medicine, Stanford, CA 94305, USA; estice@stanford.edu

**Keywords:** obesity, eating disorder risk, food reward, fMRI

## Abstract

Objective: To test whether overweight/obesity is associated with an elevated reward region response to milkshake cues and a low reward region response to milkshake receipt. To test whether the risk for eating pathology moderates the effects of weight status on the neural response to milkshake cues and milkshake receipt. Method: The current study used functional magnetic resonance imaging (fMRI) to examine the neuronal responses of female adolescents (n = 80; *M* age = 14.6 ± 0.9; *M* BMI = 21.9 ± 3.6; 41% with a biological parental history of eating pathology) during a food receipt paradigm. Results: Females with overweight/obesity showed a greater ventromedial prefrontal cortex (vmPFC), and ventral anterior cingulate (ACC) response to milkshake cues and a greater ventral striatum, subgenual ACC, and dorsomedial prefrontal cortex response to milkshake receipt than those with a healthy weight. Females with overweight/obesity plus a parental history of eating pathology showed a greater vmPFC/medial orbitofrontal cortex response to milkshake cues than those without a parental history of eating pathology and those with a healthy weight. Females with overweight/obesity and without a parental history of eating pathology showed a greater thalamus and striatum response to milkshake receipt. Conclusions: Overweight/obesity is associated with an elevated reward region response to palatable food cues and food receipt. A risk for eating pathology enhances the reward region response to food cues in those with excess weight.

## 1. Relation of Overweight/Obesity to Reward Region Response to Food Reward and the Moderating Effects of Parental History of Eating Pathology

Obesity and overweight account for over 3.4 million deaths annually [1]. Yet, most treatments do not result in long-term weight loss [2], and obesity prevention programs have shown limited efficacy [3]. An improved understanding of the differences in neural responsivity to food stimuli among individuals with overweight or obesity versus those without may guide the design of more effective obesity prevention programs and treatments.

Several theoretical models regarding neural responsivity in obesity have focused on the role of the brain’s reward circuitry. Previous studies have found that high-calorie food intake results in striatal dopamine release, that striatal dopamine release correlates positively with food pleasantness ratings, and that high-calorie versus low-calorie food tastes result in a greater reward region (striatum, medial orbitofrontal cortex [mOFC]) response [4,5,6]. According to the dynamic vulnerability theory [7], individuals at risk for obesity show elevated neural activation in reward regions (e.g., striatum) in response to the tastes of palatable, high-calorie foods, which results in overconsumption of these foods and excess weight gain. Over time, the frequent intake of high-calorie foods is thought to increase the reward region response to cues associated with these foods and cause a decrease in the reward region response to high-calorie food tastes, which increases the risk for further overeating and additional weight gain [7].

Neuroimaging studies have found that individuals with overweight/obesity show greater reward and attention region responsivity (striatum, OFC, anterior cingulate cortex [ACC]) to palatable food images and food cues [8,9,10,11,12] and less reward region (striatum) responsivity to palatable food receipt than those with a healthy weight [11,13,14,15]. However, other studies have not found evidence for a greater reward region response to food images and food cues [13,16,17,18] and a blunted reward region response to food receipt in individuals with overweight/obesity versus those without [19,20,21]. Some of the inconsistent findings may be the result of the small sample sizes in many of the studies, which increase the risk for both false positive and false negative findings [22]. Therefore, the primary aim of the study is to test the hypothesis that overweight/obesity versus healthy weight is related to greater reward region responsivity to palatable food cues and less reward region responsivity to palatable food receipt in a large sample (N = 88) of female adolescents varying in body mass index (BMI) classification from healthy weight to overweight and obesity.

The mixed findings in the literature might also relate to the heterogeneous nature of overweight and obesity. Overweight and obesity are associated with a wide range of eating disorder symptoms [23,24]. Obesity and eating pathology often co-occur and share a number of genetic and psychological risk factors [25]. Eating pathology has been associated with atypical brain reward region responses to high-calorie food stimuli. For example, females with binge-eating disorder (BED) and bulimia nervosa (BN) versus healthy controls show greater responsivity in brain reward and attention regions (mOFC, ACC) to high-calorie food images and cues [26,27,28]. There is also evidence that eating pathology enhances the relation between obesity and neural response to food stimuli. One study found that adults with both obesity and BED versus weight-matched and healthy weight controls showed greater activation in the mOFC in response to food cues [26]. Further, children with overweight/obesity and current loss of control eating relative to those with only overweight/obesity showed a greater response in brain regions related to attentional processes (middle frontal gyrus, cuneus) and emotion regulation (cingulate gyrus) during milkshake receipt [29]. These findings suggest that eating pathology increases the reward region response to food cues and food intake in those with excess weight, which increases the risk for additional weight gain.

No study has tested whether the risk for eating pathology moderates the relation between weight status and neural response to food stimuli. An improved understanding of how the risk for eating pathology alters the food-related reward response in individuals with excess weight is critical because individuals who report both obesity and eating disorder symptomology show less successful weight loss and are at a greater risk for additional weight gain and the future onset of full threshold eating disorders [23]. In a high-risk design study [30], we compared female adolescents at high risk versus those at low risk for eating disorders by virtue of biological parental history of eating pathology on neural responses to milkshake cues and milkshake receipt. We found that high-risk females showed a greater striatum response to milkshake cues than those at low risk. The risk status was not associated with a differential neural response to milkshake receipt. The second aim of the current study is to examine whether a parental history of eating pathology moderates the effects of excess weight on the reward region response to milkshake cues and milkshake receipt. We hypothesized that participants with overweight/obesity plus a parental history of eating pathology would show a greater response of the reward circuitry to the milkshake cues compared to those with healthy weight and those without a parental history of eating pathology.

## 2. Materials and Methods

### 2.1. Participants and Procedures

The participants were 88 female adolescents between the ages of 13 and 16. Advertisements in newspapers, web postings, flyers, and mailings invited female adolescents and their parents from a US city (Eugene, OR, USA) to participate in a study investigating the risk factors for problematic eating behaviors in female adolescents. We recruited females because the rates of eating disorders are higher in females compared to males [31]. Participants were recruited over a period of 2 years. Females with a biological parental history of ≥6 binge-eating episodes and/or ≥6 episodes of compensatory weight-control behaviors (purging, excessive exercise, and fasting) and females without a biological parental history of binge-eating episodes and/or compensatory behaviors were recruited [30]. Female adolescents with a history of disordered eating behaviors, current psychiatric disorders (assessed with the Schedule for Affective Disorders and Schizophrenia for School Age Children—Epidemiologic Version 5; [32]), serious medical problems, current use of weight loss drugs, use of illicit drugs or psychotropic medications, a BMI > 35 (due to scanner weight constrictions), and fMRI contraindicators were excluded. Additional exclusion criteria were the use of illicit drugs or psychotropic medications as these may be related to neural reward dysfunction [33]. Participants provided written assent, and their legal guardians provided written consent. The study was conducted according to the guidelines of the Declaration of Helsinki and approved by the Institutional Review Board of the Oregon Research Institute (title: Biological Risk Factors for Onset of Binge Eating and Compensatory Behaviors Pilot; date: 29 August 2017). Eight participants were excluded due to dropout prior to the scan assessment (n = 3), not completing the scan due to feeling claustrophobic in the scanner (n = 2), and excessive motion during the scan (n = 3). Thus, the final sample included 80 female adolescents (*M* age = 14.6 ± 0.9; *M* BMI = 21.9 ± 3.6; *M* BMI z score [zBMI] = 0.4 ± 0.9) with 61 with healthy weight, 14 with overweight, and 5 with obesity. There were 33 participants with a biological parental history of eating pathology (41%). Among the overall sample, 8.8% identified as Hispanic, 81.2% identified as white, 16.3% as mixed-race, 1.3% as Asian, and 1.2% as ‘other’. Table 1 provides participants’ demographics by weight status.

### 2.2. Experimental Procedure

Participants were asked to refrain from eating or drinking caffeinated beverages for 3–4 h before scans. Prior to the scan, they reported on the last time they ate and their hunger level (Table 2). 

*Food receipt paradigm.* This paradigm assesses neural response to tastes of a chocolate milkshake and a tasteless/odorless solution and the cues signaling the delivery of these tastes [29]. The cues were a glass of milkshake and a glass of water (2 s, 30 events each). The cues were followed by a blank jitter (1–7 s) and the delivery of either 0.5 mL milkshake or tasteless solution (5 s; 30 events each). Taste delivery occurred 7–9 s after cue onset. Participants were instructed to swallow when cued (1 s). The next cue appeared 1–7 s later. Stimuli were presented during two runs (order randomized).

### 2.3. Non-fMRI Measures

*Body mass.* Body mass index (BMI; kg/m^2^) reflects height-adjusted body weight [34]. Height was measured using stadiometers, and weight was measured using digital scales, with participants wearing light clothing without shoes. BMI was converted to *z* scores using age- and sex-adjusted BMI growth curves [35]. Overweight was defined as a *z*BMI cutoff of >+1 SD, and obesity was defined as a *z*BMI cutoff of >+2 SD [35].

*Parental eating pathology.* We used an adapted version of the Eating Disorder Diagnostic Interview (EDDI) to assess parental history of eating pathology [30]. Biological parents were interviewed to determine whether one or both had a history of ≥ 6 binge-eating episodes and/or ≥ 6 compensatory behaviors. Parents with a history of binge eating reported *M* = 22.8 binge episodes, and parents with a history of compensatory behaviors reported *M* = 42.9 compensatory behaviors during a 3-month period of peak symptoms [30]. Five percent of participants at risk for eating pathology had 2 parents with a history of ≥6 binge-eating episodes and/or ≥6 episodes of compensatory weight-control behaviors. Of the parents reporting a history of eating pathology, 81% reported compensatory behaviors without binge-eating episodes, 8% reported binge-eating episodes without compensatory behaviors, and 11% reported both binge-eating episodes and compensatory behaviors.

*Sensory and hedonic measures.* Prior to the scan, participants were asked to taste the milkshake and tasteless solution (order counterbalanced) and to rate the familiarity, intensity, pleasantness, and wanting of the tastes on cross-modal visual analog scales (VASs). The intensity of the tastes was measured using an adapted cross-modal general Labeled Magnitude Scale [36] ranging from 0 (barely detectable) to 100 (strongest imaginable sensation). Participants were also asked to rate their hunger level on a scale from 0 (I am not hungry at all) to 20 (I have never been more hungry).

### 2.4. Statistical Methods

*fMRI data preprocessing.* The data underwent preprocessing using statistical parametric mapping (SPM12; Wellcome Department of Imaging Neuroscience). The preprocessing steps included realignment, motion correction, warping, and smoothing with a 6 mm smoothing kernel using our standard approach [30]. The Artifact Detection Toolbox (ART; Gabrieli Laboratory, McGovern Institute for Brain Research, Cambridge, MA, USA) was used to detect motion and spikes in global mean response in the functional data. Head motion ≥3 mm or degrees in any direction was our a priori exclusion criterion. Motion parameters <3 mm were included as regressors in the design matrix at individual-level analysis.

*fMRI data analysis.* First-level analyses included an estimation of condition-specific effects using general linear models and convolving the canonical hemodynamic response function with stimulus events. Separate regressors modeled each condition of interest. Within participants, T-maps were constructed for comparisons of activation between the milkshake cues versus tasteless solution cues and between milkshake tastes versus tasteless solution tastes. To assess differences in the blood-oxygen-level-dependent (BOLD) response between the 2 weight status groups (i.e., obese/overweight versus healthy weight), we conducted mixed, between-/within-subjects 2 (group) × 2 (stimuli) analyses of variance (e.g., obese/overweight vs. healthy weight by milkshake receipt versus tasteless solution receipt). To assess BOLD response as a function of the interaction between weight status (obese/overweight versus healthy weight) and risk status (parental-history-positive versus parental-history-negative), we conducted mixed, between-/within-subjects 4 (group) × 2 (stimuli) analyses of variance. In addition, we conducted regression models with BMI, risk status, and the interaction between BMI and risk status. For these latter analyses, BMI was mean centered to minimize multicollinearity problems [37]. We ran whole-brain analyses using a *P*-value of <0.001 (uncorrected) in combination with a cluster-extend threshold significant at the cluster-corrected threshold of *p* < 0.05. All models included scan time and fasting (scan time minus last time eaten) as covariates of no interest. Effect sizes (*r*) for significant activations were derived from the Z-values (*Z*/√*N*).

*Exploratory analyses.* We examined the associations of the sensory and hedonic ratings of the milkshake with the BOLD response in significant clusters. For these analyses, individual-level main effect parameter estimates from significant peak coordinates were extracted from SPM and exported to SPSS to conduct the analyses. We used Bonferroni corrections to control for multiple testing.

### 2.5. Data Availability

Data and study materials can be requested from the first author. The trial that provided data was preregistered at ClinicalTrials.gov: Identifier NCT03687346.

## 3. Results

See Table 2 for descriptive statistics for the self-report measures and scan time and differences by weight status. Females with overweight/obesity showed a higher BMI (*d* = 2.75) and a higher zBMI (*d* = 2.85) and reported a lower intensity rating of the milkshake (*d* = −0.78) than those with a healthy weight (Table 2).

### 3.1. Group Differences in Neural Response to Milkshake Cues and Milkshake Receipt

**Figure 1:**
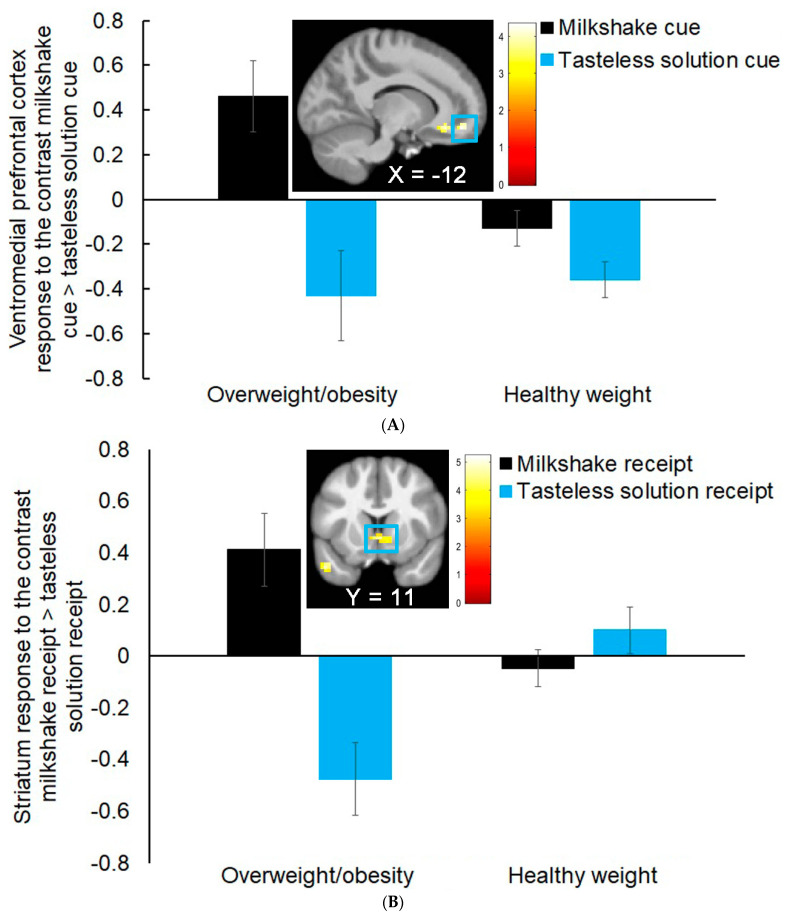
(**A**). Female adolescents with overweight/obesity versus healthy weight showed greater activation in the left vmPFC (MNI coordinates: −12, 47, −13, Z = 4.04, *k* = 33; *r* = 0.45) in response to the contrast milkshake cue > tasteless solution cue. (**B**). Females adolescents with overweight/obesity versus healthy weight showed greater activation in the right ventral striatum (MNI coordinates: 3, 11, −4, Z = 4.25, *k* = 37; *r* = 0.48) in response to the contrast milkshake receipt > tasteless solution receipt.

Females with overweight/obesity compared to those with healthy weight showed a greater left ventromedial prefrontal cortex (vmPFC *r* = 0.47; Figure 1A) and left ventral ACC (*r* = 0.46) response to the milkshake cue > tasteless solution cue contrast (Table 3). Females with healthy weight versus those with overweight/obesity did not show significantly greater neural activation in response to the contrast milkshake cue > tasteless solution cue.

Females with overweight/obesity versus healthy weight showed greater activation in the right ventral striatum (*r* = 0.48; Figure 1B), the right subgenual ACC (*r* = 0.45), and the right dorsomedial prefrontal cortex (dmPFC *r*’s = 0.38 and 0.46) in response to the contrast milkshake receipt > tasteless solution receipt (Table 3). Females with healthy weight compared to those with overweight/obesity did not show significantly greater neural activation in response to the contrast milkshake receipt > tasteless solution receipt.

### 3.2. Interaction between Weight Status and Risk Status on Neural Response to Milkshake Cues and Milkshake Receipt

In total, there were 8 high-risk (parental history of eating pathology) females with overweight/obesity, 11 low-risk (no parental history of eating pathology) females with overweight/obesity, 25 high-risk females with healthy weight, and 36 low-risk females with healthy weight (Table 1).

**Figure 2:**
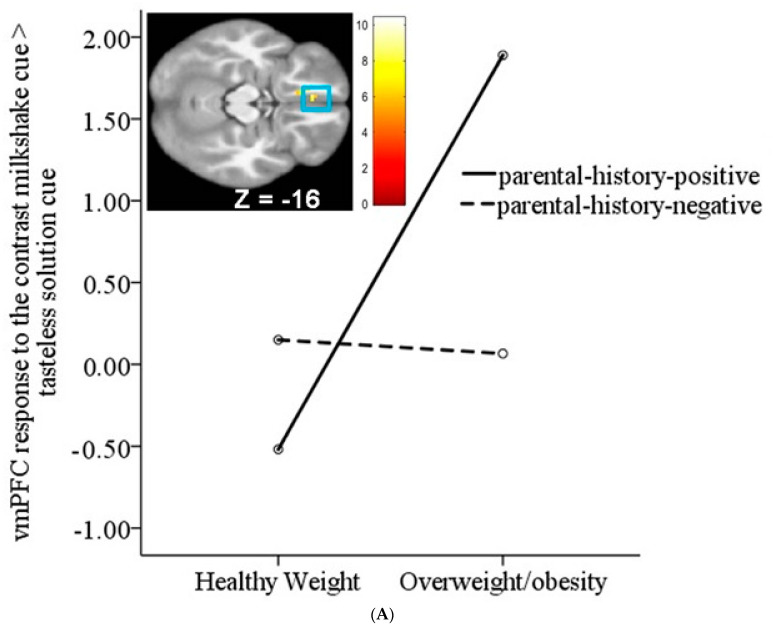

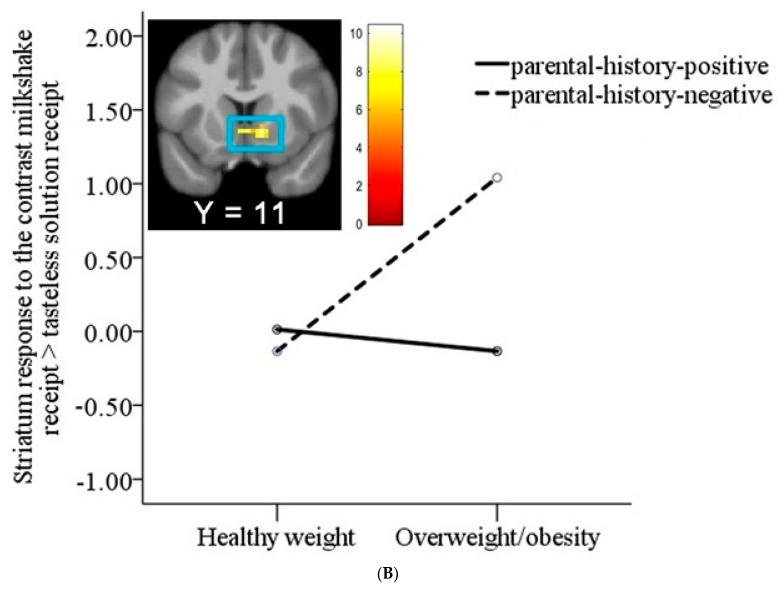
(**A**). Significant interaction between weight status and risk for eating pathology on neural response in the left vmPFC/mOFC (MNI coordinates: −6, 35, −16, Z = 3.93, *k* = 35; *r* = 0.44) to the contrast milkshake cue > tasteless solution cue. Females with overweight/obesity plus a parental history of eating pathology showed significantly greater response in this region compared to the other groups. (**B**). Significant interaction between weight status and risk for eating pathology on neural response in the right striatum (MNI coordinates: 12, 11, −4, Z = 4.68; *r* = 0.52) to the contrast milkshake receipt > tasteless solution receipt. Females with overweight/obesity and without a parental history of eating pathology showed significantly greater response in this region compared to the other groups.

ANOVA models showed that the risk status significantly moderated the effects of weight status on neural activation in the left middle frontal gyrus (MFG *r* = 0.51) and left vmPFC/mOFC (*r*’s = 0.42–0.44) (Figure 2A; Table 4) in response to the contrast milkshake cue > tasteless solution cue. Follow-up analyses showed that high-risk females with overweight/obesity showed a significantly greater response in the MFG and vmPFC/mOFC compared to low-risk females with overweight/obesity (MFG *r* = 0.47; vmPFC/mOFC *r* = 0.48), high-risk females with healthy weight (MFG *r* = 0.56; vmPFC/mOFC *r* = 0.58), and low-risk females with healthy weight (MFG *r* = 0.57; vmPFC/mOFC *r* = 0.45). There was also a significant interaction between risk status and weight status on the neural response to the contrast milkshake receipt > tasteless solution receipt in the bilateral thalamus (*r*’s = 0.49 and 0.54) and the right striatum (*r* = 0.53; Figure 2B) (Table 4). Follow-up analyses showed that low-risk females with overweight/obesity showed significantly greater responses in the thalamus and striatum compared to high-risk females with overweight/obesity (thalamus *r* = 0.64; striatum *r* = 0.70), high-risk females with healthy weight (thalamus *r* = 0.53; striatum *r* = 0.68), and low-risk females with healthy weight (thalamus *r* = 0.54; striatum *r* = 0.63).

Regression models did not show significant interaction effects of risk for eating pathology and BMI on neural activation to the contrast milkshake cue > tasteless solution cue and milkshake receipt > tasteless solution receipt.

### 3.3. Associations between Sensory and Hedonic Ratings and Neural Response to Milkshake Cues and Milkshake Receipt

Exploratory analyses examined associations between the sensory and hedonic ratings of the milkshake taste and the significant peak coordinates. No significant correlations were found and the effects were small (*r*’s < 0.25).

## 4. Discussion

Female adolescents with overweight/obesity showed greater activation in the vmPFC and ventral ACC in response to milkshake cues. The vmPFC and ACC have been found to be responsive to rewarding stimuli and are thought to be involved in the evaluation of choice options during decision making [38,39]. The vmPFC correlates with the pleasantness ratings of food stimuli [4,40] and plays a critical role in food craving [41]. The ventral ACC has been found to be involved in emotional processing and has connections with brain reward regions [42]. Our results replicate previous evidence that overweight/obesity is associated with an elevated vmPFC and ACC response to food cues [11,43] and food images [10,12,44]. Overall, these findings support the hypothesis that individuals with excess weight show hyperresponsivity in brain reward regions to palatable, high-calorie food cues, which increases the risk for overeating and additional weight gain.

Participants with overweight/obesity versus healthy weight showed a greater ventral striatum, subgenual ACC, and dmPFC response to milkshake receipt. The ventral striatum has been found to be involved in reward valuation [38,45]. The subgenual ACC correlates with motivational behavior and is thought to be involved in integrating metabolic states with the hedonic drives of food intake [46]. Further, prior work has shown that the subgenual ACC is activated during palatable food tastes and correlates positively with taste pleasantness ratings [47]. The dmPFC is thought to be involved in the cognitive evaluation of food stimuli [48]. Prior research has found that the dmPFC response to high-calorie versus low-calorie food images is greater during hungry versus sated states [8,48,49,50]. The results in the striatum are in contrast with previous work showing that an elevated BMI is related to a blunted striatum response to palatable food tastes [11,13,14,15]. A possible explanation for these discrepant findings is the difference in sample characteristics. Participants in the overweight/obese group were mostly overweight. Further, a BMI > 35 was an exclusion criterion, limiting the range of obesity. It has been suggested that reward sensitivity correlates positively with BMI from underweight levels to mildly obese levels but that it reduces with moderate obesity [51]. In support, post hoc analyses showed that BMI was positively related to ventral striatum response (MNI coordinates: 3, 11, −4) to the milkshake receipt > tasteless solution receipt contrast (*r* = 0.35, *p* = 0.006). It is therefore possible that individuals with overweight and mild obesity show an elevated striatal response to the intake of palatable foods, while individuals with severe obesity show a blunted reward response to these foods. Future studies should attempt to replicate these findings in larger samples with a wider weight range.

Participants with overweight/obesity reported lower milkshake intensity ratings than those with a healthy weight. These latter results converge with those from previous studies [52,53]. In addition, prior research has found that sweet taste sensitivity correlates negatively with sugar intake and total energy intake [54]. Taken together, these findings suggest an impairment in taste sensitivity in those with overweight/obesity.

The ANOVA models showed that a risk for eating pathology significantly moderated the effects of weight status. High-risk (i.e., parental history of eating pathology) females with overweight/obesity showed greater activation in the MFG and vmPFC/mOFC in response to the milkshake cues than those at low risk for eating pathology (i.e., no parental history of eating pathology) and those with a healthy weight. These latter results extend previous evidence that individuals with both obesity and BED show a greater mOFC response to food cues compared to weight-matched and normal-weight healthy controls [26]. A risk for eating pathology did not enhance the reward region response to milkshake receipt. In contrast, females with overweight/obesity but a low risk for eating pathology showed a greater response in taste (thalamus) and reward (striatum) regions to milkshake receipt compared to the other groups. The regression models suggested that there were no linear interactions between risk for eating pathology and BMI as a continuous variable on neural response to the milkshake cues and milkshake receipt.

Several limitations should be considered when interpreting the results. First, participants in the overweight/obesity group were overweight or mildly obese. It is possible that individuals with a higher BMI (BMI > 35) would have shown differential neural responses. Future studies with large samples varying in BMI classification from underweight to extreme obesity are needed to replicate the present findings. Second, the number of participants with overweight/obesity plus a parental history of eating pathology was small and may have resulted in false positive findings [22]. As a result, our findings need to be interpreted with caution. Third, the current study was conducted solely with female adolescents, which results in limitations in terms of generalizability.

Taken together, data from the current study suggest that overweight/obesity is associated with elevated reward region responsivity to palatable, high-calorie food cues and tastes. Further, a risk for eating pathology enhanced the reward region responsivity to the food cues but not taste in those with excess weight. These findings suggest that obesity prevention programs should strive to reduce the reward valuation of high-calorie food cues and tastes for optimal efficacy.

## Figures and Tables

**Table 1:** Demographic Data for Overweight/Obesity (n = 19) versus Healthy Weight (n = 61) Groups.

	Overweight/Obesity	Healthy Weight	Test Statistic	*P*-Value
Age [Mean ± SD]	14.5 ± 0.84	14.6 ± 0.93	*t*(78) = −0.62	0.54
BMI	27.0 ± 2.8	20.3 ± 2.0	*t*(78) = 11.7	0.00
zBMI	1.5 ± 0.35	0.01 ± 0.65	*t*(78) = 12.0	0.00
Risk status (%)			*χ*^2^(1,80) = 0.01	0.93
Parental history positive	42.1	41.0		
Parental history negative	57.9	59.0		
Maximum parental education (%)			*t*(78) = −1.19	0.24
Some high school	0	3.3		
High school graduate	15.8	1.6		
Some college	36.8	49.2		
College graduate	36.8	31.2		
Advanced degree	5.3	14.8		

**Table 2:** Results of *t*-tests Comparing Overweight/Obesity (n = 19) versus Healthy Weight (n = 61) Groups on Hunger, Last Time Eaten, Scan Time, and Hedonic Ratings.

	Overweight/Obesity	Healthy Weight	Test Statistic	*P*-Value
Hunger [Mean ± SD]	7.5 ± 5.0	9.0 ± 3.9	*t*(78) = −1.34	0.19
Last time eaten [Mean ± SD]	2.3 ± 1.6	3.1 ± 3.3	*t*(78) = −1.01	0.32
Scan time [Mean ± SD]	3 pm ± 1.5	2 pm ± 2.3	*t*(78) = 1.88	0.06
Milkshake (Mean ± SD)				
Pleasantness	14.3 ± 3.6	15.4 ± 2.5	*t*(78) = −1.59	0.12
Wanting	13.7 ± 2.7	14.9 ± 3.0	*t*(78) = −1.44	0.16
Familiarity	16.4 ± 3.7	15.6 ± 3.2	*t*(78) = 0.90	0.37
Intensity	3.9 ± 1.3	5.5 ± 2.6	*t*(78) = −3.39	0.00
Tasteless solution (Mean ± SD)				
Pleasantness	8.7 ± 2.2	9.5 ± 2.3	*t*(78) = −1.31	0.19
Wanting	8.0 ± 4.0	8.8 ± 3.3	*t*(78) = −0.92	0.36
Familiarity	11.7 ± 5.8	13.7 ± 6.2	*t*(78) = −1.22	0.23
Intensity	2.3 ± 1.1	2.4 ± 2.0	*t*(78) = −0.20	0.94

**Table 3:** Brain Regions Showing Activation During Milkshake Cues and Milkshake Receipt in Obesity/Overweight (n = 19) versus Healthy Weight (n = 61) Groups.

Contrasts	Cluster (*k*)	Z-Value	MNI Coordinates	Effect Sizes (r)
***Milkshake cue > tasteless solution cue***				
*Individuals with obesity/overweight > individuals with healthy weight*				
Ventromedial prefrontal cortex	33	4.04	−12, 47, −13	0.45
Ventral anterior cingulate cortex		3.92	−9, 32, −10	0.44
***Milkshake receipt > tasteless solution receipt***				
*Individuals with obesity/overweight > individuals with healthy weight*				
Ventral striatum	37	4.25	3, 11, −4	0.48
Subgenual anterior cingulate cortex		4.04	3, 26, −10	0.45
Dorsomedial prefrontal cortex	55	4.14	9, 65, 17	0.46
Dorsomedial prefrontal cortex		3.37	27, 56, 20	0.38

For all contrasts, activated regions, z-values, and coordinates within the MNI coordinate system are displayed. Numbers of continuous voxels (*k*) are shown for peak coordinates. For the contrast milkshake cue > tasteless solution cue, peaks within the regions were considered significant at *k* > 33, *p* < 0.05, corrected for multiple comparisons across the entire brain. For the contrast milkshake receipt > tasteless solution receipt, peaks within the regions were considered significant at *k* > 37, *p* < 0.05, corrected for multiple comparisons across the entire brain.

**Table 4:** Interactions between Weight Status and Risk Status on Neural Response to Milkshake Cues and Milkshake Receipt.

Contrasts	Cluster (*k*)	Z-Value	MNI Coordinates	Effect Sizes (r)
***Milkshake cue > tasteless solution cue***				
*Weight status X risk status*				
Middle frontal gyrus	45	4.53	−24, 26, 47	0.51
vMPFC/mOFC	35	3.93	−6, 35, −16	0.44
vMPFC/mOFC		3.81	−12, 47, −13	0.43
vMPFC/mOFC		3.74	−3, 44, −10	0.42
***Milkshake receipt > tasteless solution receipt***				
*Weight status X risk status*				
Thalamus	95	4.84	−6, −4, −1	0.54
Striatum		4.68	12, 11, −4	0.52
Thalamus		4.28	6, −4, −1	0.49

For all contrasts, activated regions, z-values, and coordinates within the MNI coordinate system are displayed. Numbers of continuous voxels (*k*) are shown for peak coordinates. For the contrast milkshake cue > tasteless solution cue, peaks within the regions were considered significant at *k* > 35, *p* < 0.05, corrected for multiple comparisons across the entire brain. For the contrast milkshake receipt > tasteless solution receipt, peaks within the regions were considered significant at *k* > 45, *p* < 0.05, corrected for multiple comparisons across the entire brain.

## Data Availability

The data presented in this study are available on request from the corresponding author.
